# Practical Application of Electrochemical Nitrate Sensor under Laboratory and Forest Nursery Conditions

**DOI:** 10.3390/s16081190

**Published:** 2016-07-28

**Authors:** William-Olivier Caron, Mohammed S. Lamhamedi, Jeff Viens, Younès Messaddeq

**Affiliations:** 1 Department of Chemistry, Laval University, Quebec, QC G1V 0A6, Canada; william-olivier.caron.1@ulaval.ca; 2 Ministère des Forêts, de la Faune et des Parcs, Direction de la recherché forestière, Quebec, QC G1P 3W8, Canada; Mohammed.Lamhamedi@mffp.gouv.qc.ca; 3 Centre for Optics, Photonics and Lasers (COPL), Laval University, Quebec, QC G1V 0A6, Canada; jean-francois.viens.3@ulaval.ca; 4 JIRU Instituto de Quimica, Araraquara-SP 14800-060, Brazil

**Keywords:** electrochemical sensor, electrochemical impedance spectroscopy, forest nursery, growing medium, nitrate

## Abstract

The reduction of nitrate leaching to ensure greater protection of groundwater quality has become a global issue. The development of new technologies for more accurate dosing of nitrates helps optimize fertilization programs. This paper presents the practical application of a newly developed electrochemical sensor designed for in situ quantification of nitrate. To our knowledge, this paper is the first to report the use of electrochemical impedance to determine nitrate concentrations in growing media under forest nursery conditions. Using impedance measurements, the sensor has been tested in laboratory and compared to colorimetric measurements of the nitrate. The developed sensor has been used in water-saturated growing medium and showed good correlation to certified methods, even in samples obtained over a multi-ion fertilisation season. A linear and significant relationship was observed between the resistance and the concentration of nitrates (*R^2^* = 0.972), for a range of concentrations of nitrates. We also observed stability of the sensor after exposure of one month to the real environmental conditions of the forest nursery.

## 1. Introduction

The introduction of technology in areas like forestry, agriculture and horticulture allows producers to collect data to control and optimize their crop management [1]. Interest in tree nursery is motivated by major challenges pertaining to sustainable growth. This includes the growth of urban infrastructure and rising urban populations, which, amongst other things, are putting pressure on production and compromising efficiency in terms of agronomy, economy and environment. The need to reduce costs and resource consumption to minimize fertilizer usage in the environment and to optimize plant production worldwide has motivated the development of low-cost, online, digital sensor technologies for monitoring the concentration of ionic nutrients in growth and groundwater systems, notably nitrate, phosphate, and potassium [2]. For example, current environmental regulations in different countries do not permit groundwater nitrogen levels to exceed 10 mg (N-NO_3_ + N-NO_2_) L^−1^ [3,4]. According to the Food and Agriculture Organization of the United Nations (FAO), the world consumption of mineral fertilizer should exceed 200,500,000 t by 2018, representing a yearly growth of 1.8% since 2015. The FAO report states a similar increase for nitrogen-based fertilizer with an annual increase of 1.4% for the same time period [5].

Nowadays, the detection and identification of nutrients still rely on conventional sampling laboratory techniques, which are costly, laborious, and not always suitable for real-time monitoring in farm or large-scale industry settings. Therefore, a research challenge in this field is focused on the need to develop rapid, reliable, specific, and sensitive methods to detect and monitor these nutrients cost-effectively [6,7], while large scale analysis implies improved miniaturization, reduction of analysis time and cost, and multi-ion detection [8].

These needs also apply to precision agriculture (PA), a farming management concept based on observing, measuring and responding to field variability in crops [9]. Crops variability typically has both spatial and temporal components, which make data acquisition, processing and statistical analysis ubiquitous. To answer the requirements of such application, a new technology must have a low-cost of production, as well as application, and be able to do real-time selective quantification of nutrients. One of the most important spatiotemporal components in PA is the leaching of nitrate, which is a global challenge for all previously stated objectives. This variability had already been quantified for forest nursery productions [10] proving the importance of such an array of sensors as the key to enable better fertilization management. In regards to the development of a new technology for these applications, tree nurseries proved to be a good testing ground as they provided great control and knowledge of many environmental and physicochemical variables.

Many ion sensors or different methods have been developed in recent years to perform crop monitoring. Among these, Electrical Conductivity (EC) meters have been used extensively to measure soil salinity [11]. However, the lack of ion selectivity for this method makes it inadequate for the quantitative measurement of specific ions. In addition, EC measurement techniques such as Time Domain Reflectometry (TDR) [10,12,13] and Frequency Domain Reflectometry (FDR) relate to the propagation of a voltage pulse and measurement of the reflected wave [14]. However, they are usually power hungry and processor intensive, which are sets of attributes inappropriate for low-cost sensors. Digital sensor technology based on electrical impedance spectroscopy (EIS) is becoming a powerful tool in precision agriculture because it involves a relatively simple electrical measurement that can readily be automated and from which results may often be correlated with many complex materials variables: From mass transport of fertilizers, rates of reactions with the growing medium and local ion concentrations [15]. EIS analysis has been used to perform tasks such as corrosion monitoring [16], fuel cell analysis [17], bio-sensing [18], mineral nutrient detection in plants [19,20], breast cancer detection [21], and glucose determination [22]. This paper describes the practical application of a novel, low-cost, and portable nitrate sensor based on EIS for the determination of trace amounts of NO_3_^−^ in selected growing media used in forest nurseries. The nitrate sensor can be integrated to conventional digital microelectronics or complementary metal-oxide-semiconductor (CMOS) platforms to perform online continuous nitrate sensing, and feed data into a database for storage and analysis. To our knowledge, this paper is the first to report the use of electrochemical impedance to determine nitrate concentrations in growing media under field conditions. First, we will describe the structural design and the impedance measurement through a simplified RC system for solutions and growing media. Then, we will compare nitrates concentration determined by the electrochemical sensors and by colorimetry for a growing medium without the presence of forest seedlings. Our aim is to determine the reliability of nitrate sensors inside a growing medium that has been fertilized with all mineral nutrients, and this over a whole first growth season of white spruce (*Picea glauca* [Moench] Voss) seedlings in a forest nursery. We will confirm the stability of the sensors during one month when they were submitted to natural environmental variables in forest nursery. All measurements are performed in compliance with the International Organization for Standardization (ISO) and the International Electro-technical Commission (IEC) certifications #17025.

## 2. Materials and Methods

### 2.1. Electrochemical Sensors and Impedance Measurements

**Figure 1:**
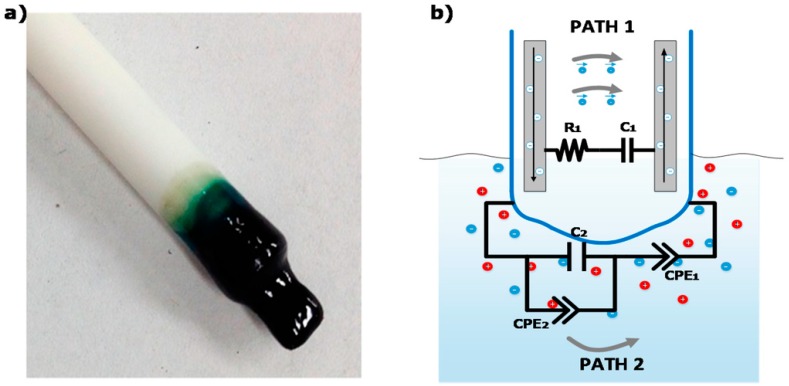
(**a**) Picture of the polyvinyl chloride-bis(2-ethylhexyl) phthalate (PVC-BEHP) electro-chemical nitrate sensor; (**b**) schematics of the main electrical conduction paths, one within the polymer membrane and the other into the medium under test (taken from Ghaffari et al. 2015) [23].

As described in our recent studies [23,24], the electrochemical nitrate sensor comprises a set of electrode wires surrounded by an ion selective polymer membrane, as shown in Figure 1a. The polymer membrane is inserted in the growing medium (preferably wet) and interacts locally with the medium under test. This sensor configuration provides two different electrical conduction paths, one within the polymer membrane and the other into the medium under test, depicted, respectively, as paths 1 and 2 in Figure 1b.

The polymer membrane is composed of high molecular weight polyvinyl chloride (PVC, from Aldrich, St. Louis, MO, USA) and of a plasticizer bis(2-ethylhexyl) phthalate (BEHP, from Aldrich). Ion-selectivity is provided by adding two components to the polymer membrane: an ionophore and an ionic site. For the nitrate sensor, the ionophore consisted of tetramethyl cyclotetra-decanato-nickel(II) complex (NiTMTAA), and the ionic site consisted of trioctylmethylammonium chloride (TOMAC, from Aldrich). Both of these have been chosen according to the reversibility, selectivity (>4 $pK_{NO_{3}^{-},A^{-}}^{pot}$, where A^−^ stands for NO_2_^−^, HPO_4_^2−^, SO_4_^2−^, or Cl^−^) and efficiency reported in previous potentiometric studies [24,25]. All chemicals, except the synthetized ionophore, were reagent grade and used without further purification. The sensors were fabricated using a dip-coating process to cover the electrodes uniformly with the polymer membrane. The electrodes were made from copper wires (923UL-9 from Consolidated Electronic Wire and Cable, Franklin Park, IL, USA), and inserted in a 5-mm-diameter, 100-mm-long alumina rod comprising two parallel hollow cavities spaced by a gap of 0.6 mm. Alumina has been chosen for its dielectric, mechanical, and chemical strength properties. The copper wires protruded out of the alumina rod of a length of 5 mm along which the polymer membranes were coated. The dip-coating process consisted of immersing the protruding copper wires into a THF-dissolved polymer solution of abovementioned composition, followed by 24-h drying at room temperature in order to let the THF evaporate and the polymer membrane solidify. Successive dipping and drying were performed until all the electrodes were fully covered by a 1-mm-thick membrane.

The sensors produced can be used on a platform that allows the measurement of complex impedance [24]. The impedance of the sensors has been obtained using an LCR-meter SR720 by Stanford Research (Sunnyvale, CA, USA). This apparatus allowed an interesting range of AC current frequency (100 Hz, 120 Hz, 1 kHz, 10 kHz and 100 kHz) and voltage (0.1 V, 0.25 V and 1.0 V) for the use of electrochemical sensors [26]. In this study, the reading was done with an AC voltage of 1 V and a frequency of 1 kHz. Those variables were chosen from the EIS results of previous study to have pseudo-linear response [23]. Even though the equivalent circuit of the sensor is known, the LCR-meter only enables to fit the impedance to simple RLC circuits. The results were extracted via the RC circuit and this system alleviates the usage of complex EIS while still extracting the real part in the electrical resistance and the imaginary part in the capacitance of the complex impedance. As it had brought more information and a more linear response to the NO_3_^−^ concentration variations, only the electrochemical resistance values will be used in this paper, the capacitance showing only important changes at high concentrations.

### 2.2. Growth Medium Selection and Nitrate Measurements

As a first step to evaluate the viability of the electrochemical sensors for applications in tree nurseries, they were used in comparison to certified measurements done on growing medium without the presence of white spruce seedlings. For this experiment, one medium was chosen for being the most used in the domain [2]. This substrate was a mix of peat-moss and vermiculite (80% V/V ratio, 0.11 g/cm^3^), which is used as a potting medium. The physicochemical properties of the two components and the mixture were characterized, as shown in Table 1.

Using this medium, certified analysis was conducted in laboratory using pure KNO_3_ solutions as an extracting agent to modify the nitrate concentration. By integrating the impedance measurement in the normalized method, it was possible to evaluate the electrochemical response of the sensor in relation to the concentration obtained by colorimetry. This protocol can be divided in three parts: Sample preparation, water content quantification, and colorimetric nitrate quantification. The goal of this procedure was to extract ionic content of a substrate into a water solution. Each sample was treated the same way three times.

Initially, this extraction was obtained by saturation of a partially dry sample using deionized water (DIW). When changing DIW using different water solutions containing nitrate, it was possible to change the nitrate content of those water-saturated samples. Those solutions were prepared in laboratory with DIW (18 MΩ·cm) and KNO_3_^−^ salt (Aldrich, selectophore grade). As the capacity exchange of the medium might change its concentration, the nitrate content presented in the paper will be the one obtained by the colorimetric measurement. Once saturated, the substrate was given time to interact with the solution, 90 ± 5 min, before being filtrated on Buchner using a whatman #4 filter. Just before this step, the sensor was put inside the substrate to do impedance measurement. Each measurement of the sensor was performed three times, allowing 120 s of conditioning time between each reading.

Once part of the sample filtrated, the filtrate was again analyzed using the electrochemical sensor. These measurements followed the same protocol of three impedance readings with the same conditioning time. Then, the filtrate was stored in a refrigerator until colorimetric analysis.

To define the water content of the saturated sample, a small quantity of it, precisely about 45 g, was dried in a stove at 110 °C overnight. The gravimetric loss of the sample represented the water content.

In a period of less than 48 h following the extraction, the filtrate must be analyzed via colorimetry. The colorimetric measurements consisted of centrifuging the filtra, passing it through a reductive column of copper-coated cadmium, and mixing it with sulfanilamide and *N*-(1-naphtyl) ethylenediamine dihydrochlorid (NED). The reduction reaction of nitrate to nitrite which reacted with sulfanilamide, forming a diazonium compound that gave a purple coloration with NED, yielding a specific optical absorption at 520 nm calibrated to provide a precise nitrate concentration.

### 2.3. Stability of the Sensor in Forest Nursery

**Figure 2:**
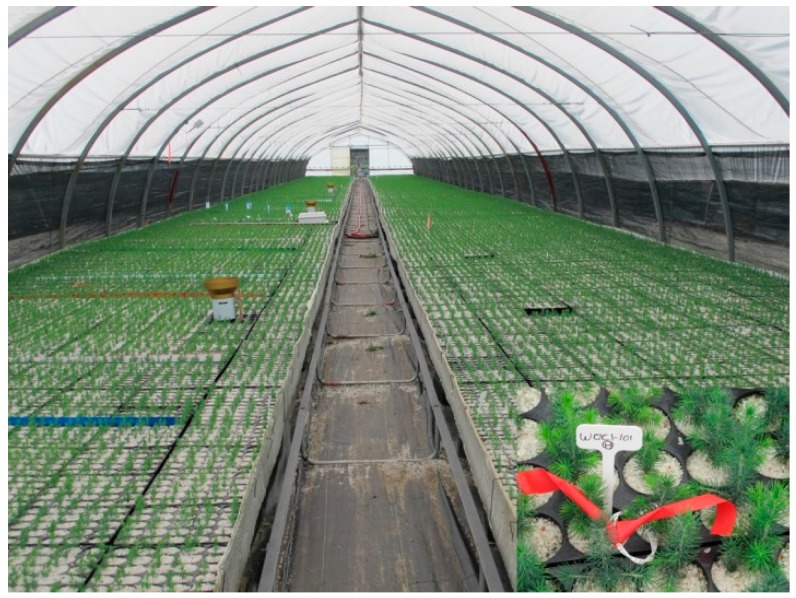
Production of white spruce seedlings in a standard production tunnel at Grandes-Piles forest nursery (Grandes-Piles, QC, Canada). Note in the corner at the bottom right, the insertion of a sensor into a cavity of 25–310 containers, in the presence of a white spruce seedling.

Three previously calibrated sensors were inserted in cavities of three randomly selected containers (model *IPL* 25–310, Saint-Damien, QC, Canada; 25 cavities, 310 cm^3^/cavity) in a production of white spruce. The cavities were filled with a moist, peat-vermiculite based substrate adjusted to a bulk density of 0.10 g/cm^3^. The cavities of all containers were covered with silica. The containers were installed in a standard production tunnel (Figure 2) at Grandes-Piles (Latitude 46°43′56″; Long. 72°42′06″), a government forest nursery located in the province of Québec.

These three sensors were subjected to a series of different environmental conditions for one month (from 16 September to 15 October 2014). The functioning and stability of the sensors were evaluated by comparing the calibration curves obtained before and after exposure of the sensors to different environmental variables.

A data acquisition system (model CR10X, Campbell Scientific, Edmonton, AB, Canada) was used to record different environmental variables inside and outside the tunnel. Temperatures in the growing medium surrounding the roots and at the substrate surface were continuously monitored (soil temperature probe model 107B, Campbell Scientific) under unheated tunnel conditions. Air temperature and relative air humidity inside the tunnel at 2.0 m above the ground surface were measured with a Vaisala RH and Temperature Probe (model HMP35C, Campbell Scientific). Three rain gauges (Model TE525M, Campbell Scientific) were used to monitor the quantity of water applied to plants during fertilization and irrigation. Light intensity was also measured continuously (model quantum sensor, Campbell Scientific).

Several details regarding the production of white spruce seedlings related to irrigation, fertilization and different cultivation techniques are described in detail in previous publications [10,12].

White spruce seedlings were irrigated and fertilised using a mechanized boom (Aquaboom, Industrie Harnois, Saint-Thomas-de-Joliette, QC, Canada) in order to eliminate the spatial variability effects of substrate water content [10]. The coefficient of uniformity of the boom is between 95% and 98%. The substrate water content was adjusted in accordance with seedling phenology (growing stage) during the first growing season (1 + 0), then maintained between 40% and 45% (V/V) [10,12]. The fertilisation regime was adjusted bi-weekly with the aid of *PLANTEC* software [27]. At the end of the first growing season (1 + 0), each seedling had received 47.28 mg of N (24.86 mg N-NH_4_, 21.22 mg N-NO_3_, 1.19 mg N-Urea), 10.64 mg of P, 15.19 mg of K and 0.09 mg of Mg, as well as micronutrients (Mn, Cu, Fe and B). Fertilisers were applied in accordance with the growing stage of the seedlings between 9 June and 29 September 2014.

### 2.4. Comparison of Determination of Nitrates by the Sensor and the Certified Laboratory Methods during the First Growing Season of White Spruce Seedlings in Forest Nursery

During the growing season, every two weeks, samples were collected and both seedling tissue and substrate were analyzed to determine their nutritional status. For each week, seedling needs for macronutrients and micronutrients were calculated depending on nutrient concentrations and the growth of white spruce seedlings according to Plantec software [27].

For this experiment and for determining the fertility of growth substrate in forest nursery including nitrates, only five sampling dates were selected (8, 11 August and 22 September and 27 October 2014). For each sampling date, five containers were randomly selected and gently extracted, for a total of 100 seedlings/date. Substrate fertility (N-NO_3_, N-NH_4_, Nmineral, P, K, Ca, and Mg), pH(H_2_O) and EC were determined on one composite sample from each container (20 root plugs/composite sample) on each sampling date. The same experimental procedure is performed on these samples.

Those samples had been taken from the same type of containers as those used in environmental stability tests and had been sampled every two weeks over the summer. Table 2 presents the sampling dates and the physicochemical properties obtained at reception in the certified laboratory. Between this analysis and the one taken with the sensors, samples had been kept refrigerated.

This information shows the variability of the ionic content of the growing medium over more than two months of production. Additionally, even if this information was not quantified, the presence of bacterial organisms might have had an impact on the sensor. By using the same protocol of analysis detailed previously for the laboratory use of sensors into the certified method of nitrate colorimetric measurement, the effectiveness of the impedance measurement will be evaluated. Once again, the results of the sensors will be compared to the ones obtained using colorimetry.

## 3. Results

### 3.1. Calibration of Electrochemical Nitrate Sensors in Solutions with LCR-Meter

Three electrochemical sensors were prepared from the same nitrate selective membrane solution composed of 60%, 30%, 7% and 3% M/M of PVC, BEHP, Ni(II)TMTAA and TOMAC respectively. 300 mg of membrane was solubilised in 3 mL of THF prior to the dip-coating process.

**Figure 3:**
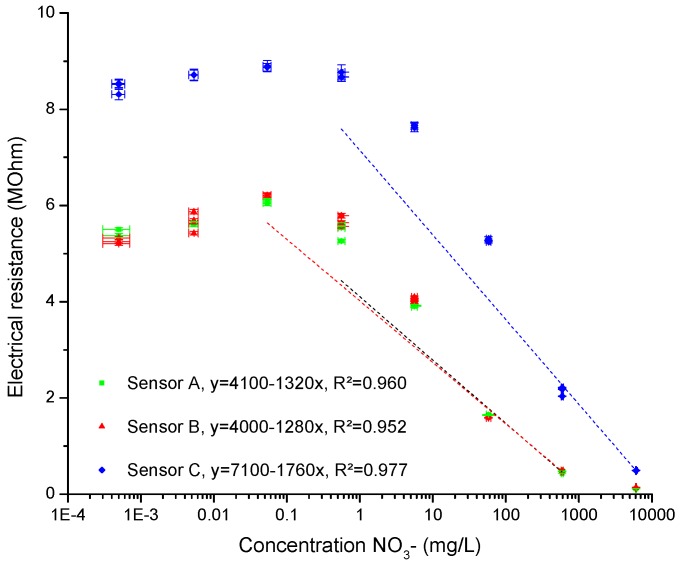
Electrochemical sensor calibration curves using KNO_3_ solutions. Each sensor presents a fitting that covers the linear portion of the signal and that is assigned by color.

After drying, each sensor was calibrated using a KNO_3_ solution covering a large selection of concentrations (10^−8^ to 10^−1^ mol/L). For the sake of continuity and comparability, the concentration values will be given in mg/L of NO_3_^−^. Every sensor read the impedance in each solution three times. The resulting resistance measurements obtained using the LCR-meter are shown in Figure 3. The fitting of a linear regression was done over the linear response range. The fitting curve will be used for further measurements.

The three sensors showed about the same range of linear response, which covers the concentration expected from the growing medium to analyse. Another important point about those three curves is that one of them had a different range of covered resistance. Additionally, a raise in resistance can be observed at low concentration. This increase has been attributed to the formation of a bilayer at the surface of the sensor, which reduces the mobility of ions. However, further studies are needed to confirm this hypothesis.

### 3.2. Nitrate Quantification in Growing Medium with Solutions

The same sensors showed in the pure solutions tests were used in laboratory analysis of growth medium. The first tests were made in pure peat-moss using DI water as the extracting agent. However, we were unable to perform comparisons with the colorimetric techniques as this test cannot quantify concentrations below 1 mg/kg of nitrate.

**Figure 4:**
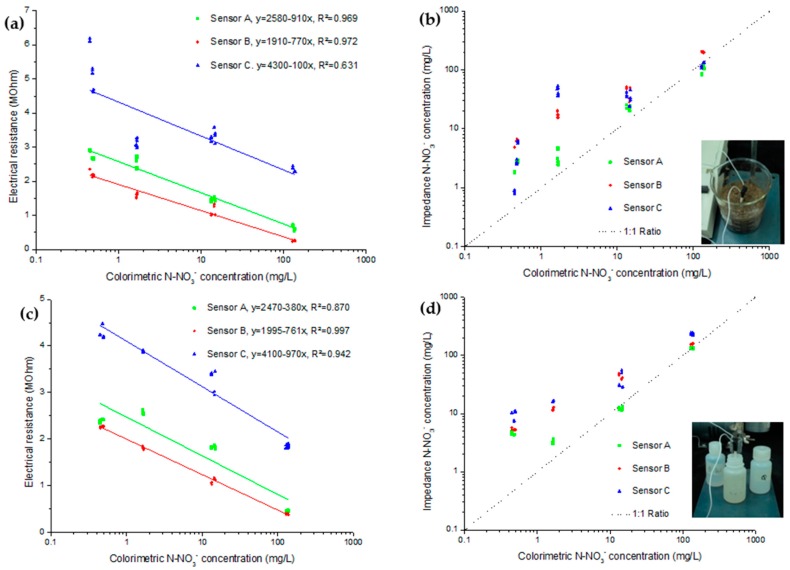
Electrochemical measurement in (**a**) impedance and (**b**) calculated concentration results obtained from saturated potting medium and in (**c**) impedance and (**d**) calculated concentration results obtained from its filtrate. Electrical resistance and calculated concentration are compared to colorimetric measurements of nitrate concentration.

The lowest concentration shown in Figure 4 is obtained using DI water. From that value, higher concentrations of the same KNO_3_ solutions used for calibration were used as the extracting agent. The selected concentrations were 10^−4^, 10^−3^ and 10^−2^ mol/L. The results of both the saturated substrate and the filtrate are shown as electrochemical measurements in function of the colorimetric measurement. It is to be noted that the difference between Table 1 data and Figure 3 concentrations is mostly attributed to the distinction of N-NO_3_^−^ in the solution stated in mg/L and N-NO_3_^−^ for dried medium stated in mg/kg. As the saturated medium had approximately a water content of 92%, the solution concentration is about ten times lower.

Even though results show a good correlation between both methods, two clear deviations can be observed. First, there is a difference between each of the three sensors in the two impedance analyses, inside the saturated substrate and inside the filtrate. The second is the different linearity between the two sets of impedance results. As the electrochemical readings from the saturated substrates showed better correlation at low concentration, these conditions will be used for further measurements in growing medium, as they will also use DI water extraction. Moreover, these conditions are more alike to in situ measurements.

### 3.3. Nitrate Quantification in Multi-Ion Fertilized Growing Medium

**Figure 5:**
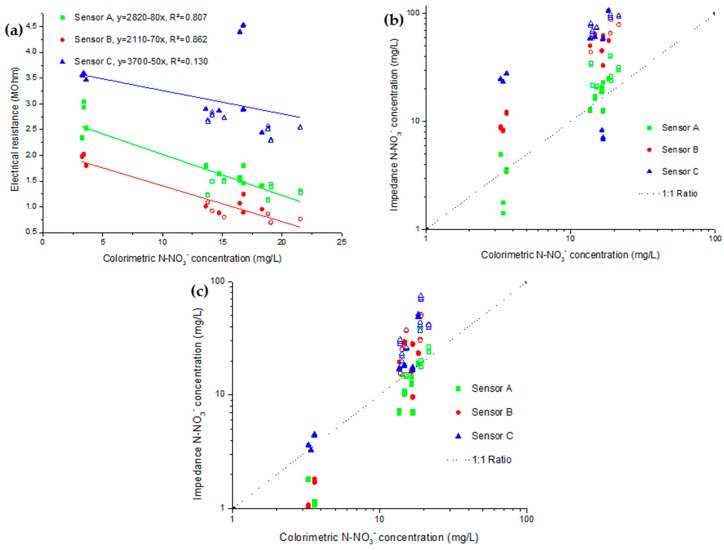
(**a**) Quantification of fertilization season samples by impedance measurement. Each sensor was associated to a linear regression to illustrate the linearity of the association; (**b**) illustrates the nitrate concentration calculated from the fitting obtained in solution as (**c**) is obtained using the fitting obtained in nitrate-doped potting medium.

By using the previously presented calibration curves, the one in solution and the saturated substrate for each sensor, quantification of nitrate was tested on growing medium sampled from forest nursery over the summer. The impedance readings were done in saturated substrate where DI water was used as the extracting agent. Even if the nitrate concentration of each medium is known for the sampling date, this information is only reliable for the samples that were refrigerated below the freezing point as more than six months had gone by between the two readings. For this reason, the concentrations obtained using colorimetric measurements were used as reference to evaluate the efficiency of the electrochemical sensors. The results of this experiment are in Figure 5. The highlighted concentrations represented with empty dots are the samples kept under the freezing point [28].

The curve of electrical resistance for the colorimetric nitrate measurement of concentration showed good linear correlation except for sensor C. As for the best fitting related to choosing the good calibration, only sensor A seemed to be close to a good ratio using the solution calibration (Figure 4b). With that calibration, sensor B and C had over-evaluated the nitrate concentration. With the calibration obtained in the saturated substrate (Figure 4c), sensors A and B under evaluated low nitrate concentrations. For the higher concentrations they kept a good correlation. In that scenario, sensor C showed the best tendency. Overall, the calibration obtained in the saturated substrate offered better results as variability at low concentration is quite small considering that the results are presented logarithmically.

### 3.4. Evaluation of Sensors Stability under Forest Nursery Conditions and Variability

**Figure 6:**
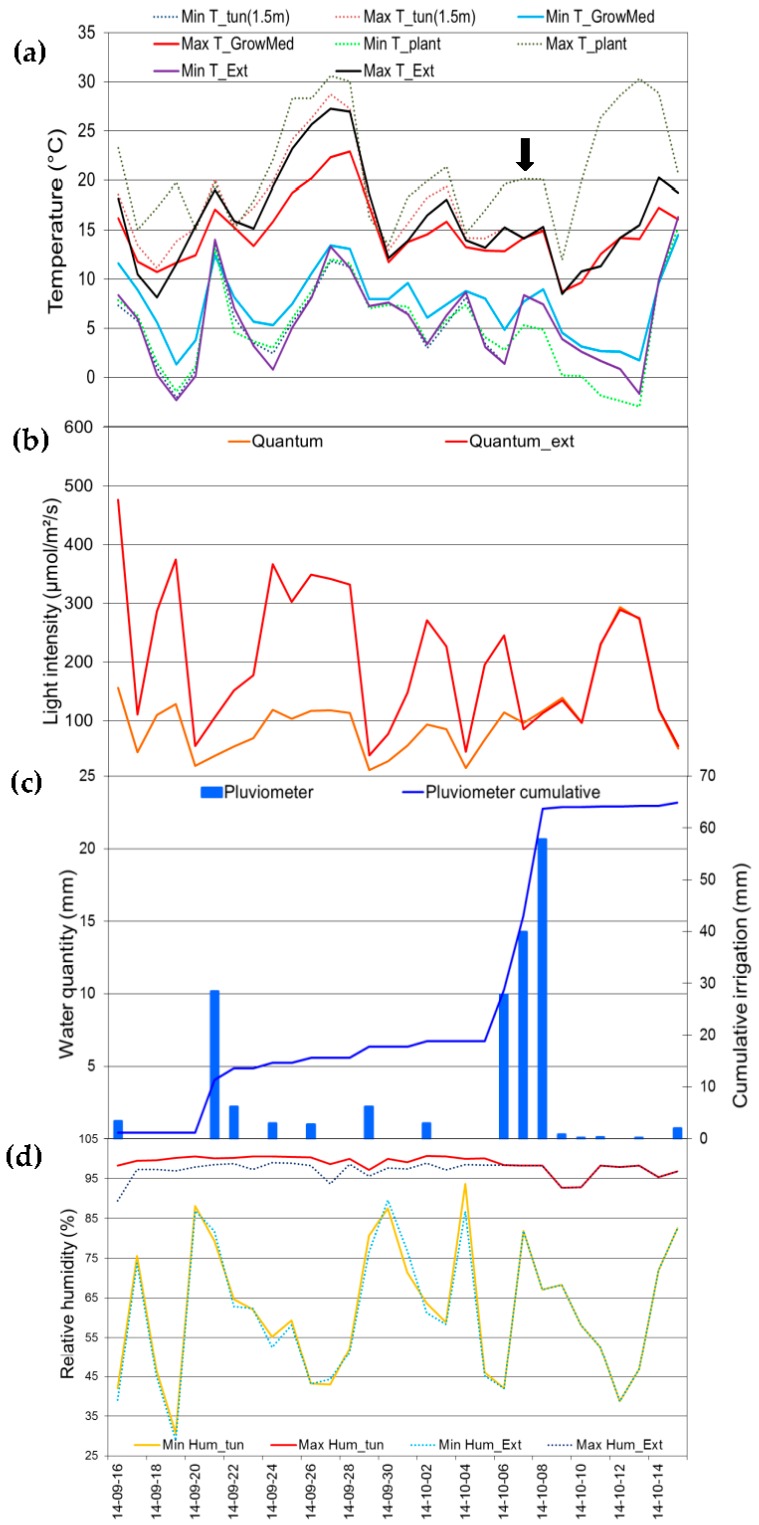
Environmental data covering the exposition time of the sensors to forest nursery conditions including (**a**) maximum (Max T_) and minimum (Min T_) temperature inside (tun(1.5 m)) and outside (Ext) the tunnel, and at the surface (plant) and in the rhizosphere of the substrate (GrowMed); (**b**) the light exposition inside (Quantum) and outside (Quantum_ext) of the tunnel; (**c**) irrigation on a daily basis and cumulative; and (**d**) minimum (Min Hum_) and maximum (Max Hum_) relative humidity for inside (tun) and outside (Ext) the tunnel. The black arrow indicates the date of the removal of the tunnel coverage corroborated by the superposition of many data points from inside and outside.

To have a good assessment of the conditions and variability to which the sensors were exposed, the data from the meteorological station was collected and studied. Over the course of the exposition, an irrigation of up to 21 mm was applied to the growing medium, reaching a cumulative value of about 65 mm. For the temperature, the environmental temperature every day reached from 27 °C to −2 °C that translates to 23 °C and 2 °C inside the substrate. The overall profile of environmental conditions is presented in Figure 6.

**Figure 7:**
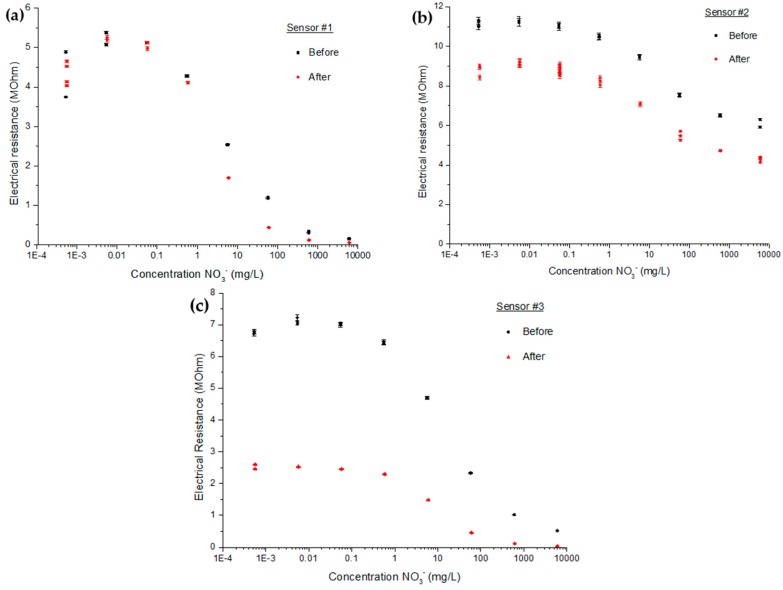
Calibration curves obtained by pure KNO_3_ solutions before and after a month of exposition to medium under forest nursery environmental conditions. The sensors are addressed as (**a**) sensor #1; (**b**) sensor #2; and (**c**) sensor #3.

For this test, three new sensors were produced using the same recipe of 60%/30%/7%/3% M/M of each respective membrane elements. The calibration curves of each of those electrochemical sensors before and after a month of exposition to these conditions are plotted over a wide range of NO_3_^−^ containing solution (Figure 7).

From the three sets of curves obtained in this experiment, it is possible to see three types of change. One sensor (#1) has nearly not changed while another one (#2) kept the same profile of response even though it had an overall drop in value. That change can be easily accounted for by a 1-point calibration without compromising the sensor efficiency. The more concerning one (#3) showed a radical change over the month of exposition by an alteration to flexion points that affect both the linearity range in concentration and in resistance. These changes did affect drastically the efficiency of the sensor and its calibration.

## 4. Discussion

We showed that the sensors can be used in pure KNO_3_ solutions by reading impedance through an LCR-meter calibrated at 1 V, 1 kHz and RC series circuit. The concentration covered by the linear response of the electrical resistance measurement is more than enough for the nitrate concentration in growing medium, starting below 1 mg/L and going above 100 mg/L [2]. The correlation between the resistance and the concentration is high enough for such a new technology produced at a laboratory level, reaching out to an *R^2^* of 0.98.

The efficiency of this setting is a good step toward the application of the device for in situ precision measurements of nitrate for fertilisation analysis. Moreover, the size and the overall price can be brought down to more cost effective components. First, this includes the scale-up of the sensor production to commercial levels, the laboratory production of each one being of about $5 at the laboratory level. Secondly, smaller hand-held devices analog to the LCR-meter are already on the market for a more affordable price, such as Smart Tweezers ST-5S from Siborg system Inc. (Waterloo, ON, Canada) [29]. This is a very important factor, as the need in the domain is a high volume of data acquisitions to enable a good mapping of spatial and temporal variabilities of a production field. These applications include the optimization of fertilizer applications to reduce nutrient leaching in agriculture, forestry and horticulture, but also for watershed management, groundwater protection, etc. Such a small digital device can also be integrated to other analytical apparatus that already reported in published paper for agriculture application [30,31].

Still, it is possible to stipulate about some limitations of the sensors. First, from one sensor to another, the impedance reading showed great variability that can be assessed directly from the calibration curve. This variability can be attributed to the production process, more precisely to the dip-coating part of the process. As this step is still hand-made at the laboratory level, the thickness and homogeneity of the membrane can vary greatly. For example, the overall rise in electrochemical resistance of a sensor compared to another one (i.e., sensor C compared to A and B) is associated to a thicker membrane surrounding the electrodes. As there is non-destructive way to assess the thickness, it was impossible to define precisely the impact of this factor.

Another major variability stood in the results obtained directly inside tree nursery conditions. These variations can be related to the production process of the different sensors. Additionally, the aging process may explain such disparity in the reported values. Additionally, the position variability of the sensor in relation of the container and to the plant growing in it, especially in relation to the roots or high mineral content particles like vermiculite. Even so, the fact remains that one of the sensors showed nearly no change through its exposition. This suggested that the stability of the sensor is achieved by the current design and could be usable for in situ measurements.

Homogeneity of tree nursery environmental conditions was not the only result that showed the electrochemical sensors usability for in situ fertilisation measurements. The fact that LCR-meter readings in the saturated substrate showed similar and even better results than in the filtrate, brings more a direct usability of sensors in tree nurseries. This difference was associated to the anionic exchange capacity which, even if low, allowed a good stability of nitrate ions in solution inside the water content of the saturated medium. That intrinsic selectivity coming from the growing medium affected the ionic content kinetically by the presence of peat-moss and vermiculite particles.

Though this selectivity gained by chemical interaction with particles was an advantage, the results showed that it was not enough to specifically read nitrate variations through the fertilisation season. This can be assumed from the difference between colorimetric results and electrochemical results obtained on substrate sampled over the fertilisation season. The fact that for the same calibration, nitrate concentrations had been mostly over-evaluated in this experiment, it’s an indication that the rest of the ionic content, presented in Table 2, is part of the impedance reading. Still, the impedance results from this experiment showed a relation between the full ionic content and electrical resistance.

Overall, even though the nitrate sensor was unable to achieve a selective reading of the NO_3_^−^ content of a growing medium, it had achieved quantitative measurements inside a pure KNO_3_ water solution and a pure KNO_3_ doped growing media. As stated in the previous article, this can be attributed to the design limitation of the sensors related to the conductivity pathway chosen by the current over the ionic concentration in the solution [24].

## 5. Conclusions

We present the first glimpse of a practical application of a new electrochemical sensor based on electrochemical impedance spectroscopy in a controlled tree nursery environment and in a certified growing medium analysis. In this regard, the presented measurements of electrical resistance using RC series circuit of an LCR-meter for concentrations ranging from 0.1 mg/L to 1000 mg/L of nitrate; show a linear response wide enough to cover the needs of the domain of nitrate quantification in growing medium. The sensors also showed the possibility of a great stability for over a month of environmental exposition inside tree containers. Moreover, even if the sensor did not show the expected nitrate selectivity related to the chosen ion selective membrane, the impedance measurement using the LCR-meter apparatus proved to be effective in the pure nitrate containing water solution and the nitrate-doped saturated growing medium. In addition, the overall ionic content can be quantified by the use of the sensors, which enable a crude in situ analysis to follow the impact of fertilisation to this system.

Moreover, through the experiment, the size and the format of the actual design was a perfect fit to forest nursery containers. Combined with the ability to do quantitative measurements in growing medium, it is now possible to think about a new generation of sensors that will give a better control of both the fabrication process and the accessible current pathway. The main challenge of this second generation device will be to achieve selectivity.

## Figures and Tables

**Table 1:** Physicochemical parameters about selected potting medium and its components. The data were obtained under analysis at an ISO-17025 certified laboratory. They are the average of three samples of each type.

Parameter	Peat-Moss	Vermiculite	Potting Medium
pH-H_2_O	4.27	6.55	3.80
pH-CaCl_2_	2.85	4.81	3.10
Electrical Conductivity (µS/cm)	68.1	10.9	173
N-NH_4_^+^ (mg/kg)	41	<1	40
N-NO_2_^−^ + NO_3_^−^ (mg/kg)	2	<1	6
P (mg/kg)	2	<1	13
K (mg/kg)	11	4	20
Ca (mg/kg)	13	2	29
Mg (mg/kg)	5	3	45
Humidity-Sat. (%)	91.5	79.9	88.15

**Table 2:** Physicochemical parameters about sampled growth medium identified by sampling period. The data were obtained under analysis at an ISO-17025 certified laboratory. * Those samples were kept refrigerated below 0 °C.

Parameter	* 11 August 2014	* 8 September 2014	22 September 2014	6 October 2014	27 October 2014
pH-H_2_O	4.27	4.39	4.38	4.45	5.05
Conductivity (µS/cm)	219	211	171	147	21.4
N-NH_4_^+^ (mg/kg)	88	71	44	38	7
N-NO_2_^−^ + NO_3_^−^ (mg/kg)	150	187	130	123	2
P (mg/kg)	34	42	61	56	13
K (mg/kg)	61	66	47	37	10
Ca (mg/kg)	37	41	35	23	10
Mg (mg/kg)	94	107	90	235	13
Mn (mg/kg)	1	1	<1	<1	<1
Cu (mg/kg)	<1	<1	<1	<1	<1
Zn (mg/kg)	<1	<1	<1	<1	<1
Al (mg/kg)	4	5	5	3	6
Fe (mg/kg)	5	7	8	7	7
Mo (mg/kg)	< 1	< 1	< 1	< 1	< 1
Na (mg/kg)	25	32	31	30	12
B (mg/kg)	1	1	1	3	<1
S (mg/kg)	78	46	40	26	9
Humidity-Sat. (%)	91.54	91.78	91.46	91.76	92.16
